# Editorial: Benefits and challenges to using health-related information and communication technologies among older adults

**DOI:** 10.3389/fpubh.2023.1224606

**Published:** 2023-06-13

**Authors:** Ronald W. Berkowsky, Alexander Seifert, Timothy M. Hale

**Affiliations:** ^1^Health Sciences Program, California State University Channel Islands, Camarillo, CA, United States; ^2^School of Social Work, University of Applied Sciences Northwestern Switzerland, Olten, Switzerland; ^3^Department of Kinesiology and Community Health, University of Illinois Urbana-Champaign, Champaign, IL, United States

**Keywords:** older adults, elders, aging, information and communication technologies, ICT, health technology, digital divide, human-computer interaction

To accommodate the current and anticipated cost and care demands of a rapidly aging global society (1), relevant stakeholders in public health and clinical care have turned toward health-related information and communication technologies (ICTs) to help mitigate these growing issues (2). Internet-connected devices and their associated programs and applications have been shown to increase digitally-supported healthcare access among older adults, such as through expanding and enhancing interactions with healthcare providers via telehealth during the COVID-19 pandemic (3, 4). ICTs have also been shown to assist older patients in managing or mitigating physical (5), mental (6), and social (7) health and wellbeing issues. Despite these known benefits, and despite trends indicating that overall use of ICTs among older adults is increasing (8), many still encounter significant barriers to access and use of health-related ICTs (9). In this Research Topic, scholars from across the globe provide theoretical insight and current empirical findings on both the benefits and barriers associated with health-related ICT use among older adults. This work frames ICT use among older adults as a public health issue—that, in addressing and eliminating the barriers to health-related ICT use, older adults will benefit physically, mentally, and socially.

Three manuscripts in the collection specifically examine the potential impacts of ICT use among older adults. He et al. examine the potentially mitigating effects of Internet use in the relationship between visual impairment and depressive symptomatology. They find that Internet use is negatively associated with depression among Chinese middle-aged and older adults, and Internet use serves as a significant mediator between visual impairment and depression such that Internet use lowers the risk of depression. Li et al. examine the efficacy of a medication management intervention delivered via pharmacist-led telemedicine among patients with hypertension during COVID-19, finding that telemedicine can improve patient outcomes and increase medication adherence—this adds to a growing body of literature underscoring the value of digital care delivered across various specialties and disciplines (10). Finally, building on other's work examining the relationship between ICT use and mental health (11), Schuster et al. investigate the potentially bi-directional relationship between online health information-seeking and anxiety among older adults using longitudinal data. Interestingly, they find that while elevated levels of anxiety may contribute to elevated levels of online health-information seeking behavior, it does not appear that changes in online health information-seeking significantly impacts anxiety.

A majority of the studies in the collection highlight barriers to use of health-related ICTs among older adults, with many focusing on factors related to willingness and readiness to adopt ICTs. Jokisch et al. expand upon previous work examining factors predicting technology adoption among older adults, finding that perceived usefulness, self-efficacy in utilizing digital technologies, privacy/security concerns, support from family, and support from formal and institutional settings all showed significant associations with intention to use digital health services. This work supports similar studies (12) showing that perceived value and confidence in learning are significant predictors of willingness to adopt technology among older adults. The findings by Jokisch et al. were echoed in the Seinsche et al. study which examined older adult and health practitioner perspectives on use of exergames (i.e., digital games targeting physical and cognitive functions). They find that while older adults may report less motivation and interest in using general ICTs, they report higher interest in exergames due in part to their perceived value, contrary to the expectations of health practitioners. Other factors motivating use include simplicity in set-up, detailed user instructions, having more personalized (i.e., more valuable) training, and having a reachable contact for assistance and guidance. Wang and Zhao examine intention to use a different ICT (patient-accessible electronic health records, or PAEHRs)—applying concepts from ecological psychology (13), they find that multiple PAEHR characteristics, such as serving multiple functions and flexibility in navigation, significantly predict attachment to PAEHRs and doctors which in turn predicts intention to use.

Additional studies in the collection examine other predictors of technology adoption and use among the general older population. Gray and Charness examine ICT age and find that older adults are more likely to use more dated devices (i.e., non-smart cellular phones) and older desktop computers, laptop computers, and smartphones, implying that many older adults may not utilize the necessary updated ICTs to engage in certain health practices (e.g., participate in an online health intervention). Barriers to use of smart senior care devices is explored by Kong et al. via qualitative semi-structured interviews with 15 older adults in Southwest China. They find that while those interviewed recognized a value in smart care devices and found them to be relatively implementable, numerous obstacles to use were identified including a lack of awareness of the devices and their functions, technophobia, not wanting to upset living habits, and concerns with cost and security. Finally, Cao et al. contribute to the growing literature examining social capital as a positive predictor of digital health information-seeking (14) and, more specifically, eHealth literacy (15) among older adults. Interestingly, the results of this investigation contrast with findings from similar studies—social support was not found to be a significant predictor, nor was gender, underscoring the importance of identifying differences in variable measurement and in contextual factors of data collection (e.g., geographic location, cultural influences).

Two of the manuscripts in the collection identify barriers to successful use of ICTs among specific segments of the older adult population which have received less attention. In their qualitative work, van Leersum et al. examine the use of the virtual personal assistant Anne4Care among elder migrants in the Netherlands (majority Turkish), identifying various barriers (e.g., lack of trust in the technology or those that prescribe them, need for support, language barriers) but also finding that these elders found worth in the technology. Xu et al. examine the subjective experiences of deaf and hard-of-hearing Chinese elders in using digital technologies during COVID-19 and find that deaf and hard-of-hearing older adults are often barred from health and socialization benefits due to limits in digital access, less experience in using the technologies, and a lack of accommodation to their ability status—this can limit health information-seeking ability and promote isolation, further underscoring the need for adapting technologies based on need. This need—to adapt technologies to the user—is the central thesis of the final manuscript in the collection by Cevallos et al.. In it, the authors re-examine the concept of *aging in place*, referring to preferences of older adults to age in the home or in communities that promote identity, autonomy, independence, and comfortability (16), and argue for consideration of implementing continuous accommodations via ICTs into the residential space of older adults. This reconceptualization, referred to as *(st)aging in place*, argues that technologies utilized in the home should adapt to the preferences of the aging individual through the life course as their needs change.

Despite the strength of the research and diversity in topical area provided in this collection, it is not without limitations which echo criticisms levied on the broader scholarship—as an example, the studies included do not specifically examine benefits and challenges of ICT use in long-term care settings, and there is a lack of diversity in scholarship by geographic location. These limitations, along with the swiftness in which health-related ICTs update and evolve, underscore the need for continued research in this area to fully elucidate the ways in which ICTs can be positively and successfully incorporated into the daily lives and care of older adults across the globe. Future work can also more fully elucidate the ways in which public health professionals can best harness the potential of health-related ICTs in managing the care of older adults and reducing the costs associated with this care.

## Author contributions

RB was responsible for writing the initial draft of the editorial. AS and TH were responsible for reviewing and revising the draft. All authors approved the current version prior to submission.

## References

[1] Bloom DE Canning D Lubet A. Global population aging: facts, challenges, solutions & perspectives. Daedalus. (2015) 144:80–92. 10.1162/DAED_a_00332

[2] Fares N Sherratt RS Elhajj IH. Directing and orienting ICT healthcare solutions to address the needs of the aging population. Healthcare. (2021) 9:147. 10.3390/healthcare9020147 33540510 PMC7912863

[3] Kim YK Ang S. Older adults with functional limitations and their use of telehealth and during COVID-19. Res Aging. (2022). 10.1177/01640275221147642 [Epub ahead of print]. 36562247 PMC9790857

[4] Seifert A Batsis JA Smith AC. Telemedicine in long-term care facilities during and beyond COVID-19: challenges caused by the digital divide. Front Publ Health. (2020) 8:601595. 10.3389/fpubh.2020.601595 33194999 PMC7649197

[5] Schulz R Wahl HW Matthews JT De Vito Dabbs A Beach SR Czaja SJ. Advancing the aging and technology agenda in gerontology. Gerontologist. (2015) 55:724–34. 10.1093/geront/gnu071 25165042 PMC4592332

[6] Cotten SR Ford G Ford S Hale TM. Internet use and depression among retired older adults in the United States: a longitudinal analysis. J Gerontol Ser B Psychol Sci Soc Sci. (2014) 69:763–71. 10.1093/geronb/gbu018 24671896

[7] Schlomann A Seifert A Zank S Woopen C Rietz C. Use of information and communication technology (ICT) devices among the oldest-old: loneliness, anomie, and autonomy. Innov Aging. (2020) 4:igz050. 10.1093/geroni/igz050 31911953 PMC6938466

[8] Hung LY Lyons JG Wu CH. Health information technology use among older adults in the United States, 2009–2018. Curr Med Res Opin. (2020) 36:789–97. 10.1080/03007995.2020.1734782 32096650

[9] Seifert A. The digital exclusion of older adults during the COVID-19 pandemic. J Gerontol Soc Work. (2020) 63:674–6. 10.1080/01634372.2020.1764687 32401181

[10] Kruse C Fohn J Wilson N Patlan EN Zipp S Mileski M. Utilization barriers and medical outcomes commensurate with the use of telehealth among older adults: systematic review. JMIR Med Inform. (2020) 8:e20359. 10.2196/20359 32784177 PMC7450384

[11] Cotten SR Goldner M Hale TM Drentea P. The importance of type, amount, and timing of internet use for understanding psychological distress. Soc Sci Q. (2011) 92:119–39. 10.1111/j.1540-6237.2011.00760.x 21534270

[12] Berkowsky RW Sharit J Czaja SJ. Factors predicting decisions about technology adoption among older adults. Innov Aging. (2017) 1:igy002. 10.1093/geroni/igy002 30480129 PMC6177084

[13] Zhao YC Zhang Y Tang J Song S. Affordances for information practices: theorizing engagement among people, technology, and sociocultural environments. J Document. (2020) 77:229–50. 10.1108/JD-05-2020-0078

[14] Ahn S Lee CJ Ko Y. Network social capital and health information acquisition. Patient Educ Couns. (2022) 105:2923–33. 10.1016/j.pec.2022.05.007 35637049

[15] Hayat T Brainin E Neter E. With some help from my network: supplementing eHealth literacy with social ties. J Med Intern Res. (2017) 19:e98. 10.2196/jmir.6472 28360024 PMC5391437

[16] Wiles JL Leibing A Guberman N Reeve J Allen RE. The meaning of “aging in place” to older people. Gerontologist. (2012) 52:357–66. 10.1093/geront/gnr098 21983126

